# Nutritional Status of Pediatric Cancer Patients at Diagnosis and Correlations with Treatment, Clinical Outcome and the Long-Term Growth and Health of Survivors

**DOI:** 10.3390/children7110218

**Published:** 2020-11-07

**Authors:** Vassiliki Diakatou, Tonia Vassilakou

**Affiliations:** 1 Children’s & Adolescents’ Oncology Radiotherapy Department, Athens General Children’s Hospital “Pan. & Aglaia Kyriakou”, GR-11527 Athens, Greece; vdiakatou@hotmail.com; 2 Department of Public Health Policy, School of Public Health, University of West Attica, Athens University Campus, 196 Alexandras Avenue, GR-11521 Athens, Greece

**Keywords:** childhood cancer, pediatric oncology, nutritional status, malnutrition

## Abstract

Malnutrition is caused either by cancer itself or by its treatment, and affects the clinical outcome, the quality of life (QOL), and the overall survival (OS) of the patient. However, malnutrition in children with cancer should not be accepted or tolerated as an inevitable procedure at any stage of the disease. A review of the international literature from 2014 to 2019 was performed. Despite the difficulty of accurately assessing the prevalence of malnutrition, poor nutritional status has adverse effects from diagnosis to subsequent survival. Nutritional status (NS) at diagnosis relates to undernutrition, while correlations with clinical outcome are still unclear. Malnutrition adversely affects health-related quality of life (HRQOL) in children with cancer and collective evidence constantly shows poor nutritional quality in childhood cancer survivors (CCSs). Nutritional assessment and early intervention in pediatric cancer patients could minimize the side effects of treatment, improve their survival, and reduce the risk of nutritional morbidity with a positive impact on QOL, in view of the potentially manageable nature of this risk factor.

## 1. Introduction

The importance of nutrition in children with cancer is indisputable [1]. Nutrition influences most cancer control parameters in pediatric oncology, including prevention, epidemiology, biology, treatment, supportive care, recuperation, and survival [2]. It is widely recognized that the nutritional status (NS) of children diagnosed with and treated for cancer will be probably affected during the course of the disease. 

NS of pediatric cancer patients has been researched for a lengthy time and nutritional problems have long-been recognized [3,4,5,6,7,8]. Indeed, publications on childhood cancer related undernutrition have appeared since the 1970s [9], however its management remains variable [1,4,10], with many undernourished children not timely recognized and therefore not treated [11].

The importance of NS in childhood cancer patients concerns its potential impact on disease progression and survival [1]. The NS at the time of diagnosis can affect outcomes in terms of morbidity and mortality [12]. Additionally, nutrition related problems can affect the quality of life (QOL) of survivors, as well as predispose them to other chronic diseases [2]. This fact highlights the need for scientific management and nutritional support for this population.

At the same time, the available data regarding the prevalence of poor nutritional status are derived at different phases of the disease and are highly variable among diagnostic groups, as well as between developed and developing countries [1,13,14,15]. The heterogeneity of diagnoses, the different stages of treatment and the followed treatment protocols complicate any straightforward comparison among studies. Moreover, the variety of definitions for malnutrition, the methodology used to assess the NS—in terms of anthropometric measurements—as well as criteria and cut-off points, make an accurate estimation of the prevalence of cancer related malnutrition very difficult.

This review aims to identify NS alterations that occur during the management of childhood cancer. The purpose of the study is to investigate how neoplastic diseases affect the NS of children and adolescents, as well as how the nutritional profile affects treatment response, clinical outcome and long-term growth and health of survivors. By investigating the multifactorial components of nutrition in childhood cancer morbidity, this work aims to be the trigger to recognize the importance of nutrition in order to become an integral part of cancer treatment in children and adolescents in Greece.

## 2. Materials and Methods

An electronic search of the international literature was performed, using the Cochrane Library, MEDLINE, SCOPUS, and PUBMED to identify systematic reviews, meta-analyses, randomized controlled trials and observational studies published during the period 2014–2019. The search strategy identified the following keywords and medical subject heading searches (MeSH): “childhood cancer”, “pediatric oncology”, “nutritional status”, and “malnutrition”. The reference list of all relevant articles was also examined, and possibly relevant corresponding articles were hand-searched. Particular attention has been paid to most recent articles, meta-analyses and systematic reviews conducted in countries with different socioeconomic status in order to identify possible influence. Studies involving adult patients were excluded.

## 3. Results

### 3.1. Nutritional Status at Diagnosis

Many former and recent studies have investigated the issue of weight changes in children diagnosed with cancer. Pediatric cancer includes a heterogeneous group of diagnoses, while the repercussions, prognosis and the therapeutic planning differ according to tumor location, histological type, nature as well as biological behavior and age of incidence [16]. Such differences also influence the NS, in a way that some patients present with weight loss at diagnosis, thus being at higher risk for suboptimal NS during the anticancer treatment [17,18]. NS at the time of the diagnosis is an important factor which influences the response to the treatment as well as the possibility of recovery [1].

In the cross-sectional observational study of Maia Lemos et al. [16], the authors assessed the NS of 1154 children and adolescents with malignant neoplasms in Brazil at the time of diagnosis. At that time point, 67.63% of patients presented adequate body mass index (BMI). The overall prevalence of undernutrition was 10.8%, 27.3%, 24.5% and 13.6%, based on BMI, triceps skinfold thickness (TSFT), mid-upper arm circumference (MUAC), and arm muscle circumference (AMC), respectively [16].

Villanueva et al. [19] studied the NS of 1060 patients diagnosed with cancer in Guatemala. NS was evaluated by MUAC, TSFT, and serum albumin (ALB) levels. Children were nutritionally classified as adequately nourished, moderately depleted, and severely depleted. With regard to diagnoses, leukemia accounted for 51% of all diagnoses, followed by solid tumors (33%), lymphomas (11%), and brain tumors (BT) (5%). At diagnosis, 47% (*n* = 495) of patients were severely nutritionally depleted, 19% (*n* = 207) were moderately depleted, and 34% (*n* = 358) were adequately nourished.

In total, 74 pediatric oncology patients newly diagnosed with hematological malignancies (*n* = 56) or solid tumors (*n* = 18), were included in a prospective observational cohort study conducted in Istanbul [20]. Anthropometric measurements included body weight, height, BMI, BMI for age percentile, MUAC, TSFT as well as z-scores for weight for age (WFA), height for age (HFA), BMI for age, weight for height for age, MUAC for age, and TSFT for age. At diagnosis, undernutrition (BMI for age z<−2 standard deviation (SD)) was evident in nine (12.3%) of 74 patients, including six (10.9%) patients with hematological malignancies and three (16.7%) patients with solid tumors, whereas undernutrition (BMI<5th age percentile) was evident in 10 (13.7%) of 74 patients, including six (10.9%) patients with hematological malignancies and four (22.2%) patients with solid tumors. In addition, increased body weight (BMI for age z > 2 SD) was evident in five (6.8%) patients.

Pribnow et al. [21] conducted a retrospective review of newly diagnosed patients with acute lymphoblastic leukemia (ALL), acute myeloid leukemia (AML), Wilms tumor, Hodgkin lymphoma (HL), or Burkitt lymphoma (BL) in Nicaragua. A total of 473 patients were assessed and 282 patients were recruited in the study. At diagnosis weight, height or length measurements were recorded and NS assessment included BMI, MUAC, TSFT, and levels of serum albumin. At diagnosis, on the basis of NS categories, 67% of patients were undernourished, 19.1% suffered from moderate undernutrition and 47.9% were severely undernourished. Undernutrition rates were higher in patients with Wilms tumor (85.7%) and BL (75%) and lower in those with HL (58.3%). Patients with high-risk malignancies were inclined to have inferior NS regardless diagnosis, when comparing adequately nourished (37.3% of patients with high-risk disease) to severely undernourished (62.7% of those with high-risk disease) groups (*p* = 0.08). Similar trends are also observed in high-income countries (HICs) and can be attributed to disease burden.

In the same country, a cohort of 104 patients was screened for NS at diagnosis [22]. The NS assessment was based on weight, height or length and the anthropometric measures of MUAC and TSFT. Thirty-four patients were affected by ALL, five by AML, 13 by lymphomas and 52 by solid tumors, including BT (*n* = 20), retinoblastoma (*n* = 3), bone and soft-tissue sarcoma (*n* = 15), Wilms’ tumor (*n* = 7) and others (*n* = 7). Yet, diseases were clustered in two groups—leukemia/lymphomas and solid tumors—for further analyses. According to their anthropometric measurements, patients were overall classified as 65.4% severely depleted, 13.5% moderately depleted, and 21.1% borderline/adequately nourished, that is considered at risk of developing undernutrition during treatment.

In the largest study so far from India, a total of 1693 new patients were enrolled, of whom 1187 had all anthropometric measurements performed [23]. The prevalence of undernutrition—defined by World Health Organization (WHO) criteria—at the time of diagnosis was very high ranging from 40–80% depending on the method used for assessment, being higher with MUAC and lowest with BMI. Specifically, the prevalence of undernutrition was 38%, 57%, 76%, 69%, and 81% on the basis of BMI, TSFT, MUAC, AMC, and arm TSFT + MUAC, respectively. Addition of BMI and serum albumin to arm anthropometry increased the proportion classified as severely nutritionally depleted by a mere 2% and 1.5% respectively. Among disease groups, no considerable differences were found in undernutrition rates, consistent with findings of a similar large study published at that time [24]. On the other extreme, only 14 (0.8%) of children in this study were obese among the whole group, much lower than the 14% rate of obesity in a recent large study from the United States of America (USA), reflecting the socio-economic influence on NS [25].

Another study conducted in India, analyzed retrospectively weight records collected at diagnosis for patients with ALL, AML, solid tumors, and lymphomas [26]. A total of 295 pediatric patients were enrolled in the analysis. Patients’ weight was plotted on WFA growth charts of Center for Disease Control and Prevention (CDC) [27]. At diagnosis, 153 out of 295 (52%) of patients had WFA between 3rd and 97th centile and were therefore considered to be well-nourished, 130 out of 295 (44%) patients were undernourished and 12/295 (4%) patients were obese. The prevalence of undernutrition at admission among males and females was 44% and 42%, respectively. As regards the diagnosis, there was no significant difference in NS at diagnosis between hematological malignancies and solid tumors (*p* = 0.8).

NS at the time of cancer diagnosis is dependent on a cancer type, its localization and clinical stage of the disease [1]. In addition, prevalence rates of malnutrition depend not only on methods and criteria used to assess NS, timing of assessment and composition of the study population—in terms of types of malignancies—, but also on socio-economic status [3]. Studies carried out in countries with better socio-economic conditions showed different results from the above mentioned. Moreover, other factors such as poverty, lack of adequate education and health support can aggravate nutritional risk especially in developing countries [16]. In general, undernutrition rates have been found much higher in low- or low-middle-income countries (LMICs) (40–90%) in comparison to countries with high or medium income (0–30%) [4,24,28,29,30].

In the USA, a cohort of 2,008 children treated for high-risk ALL enrolled in Children’s Oncology Group study CCG–1961 (Children’s Cancer Group) [25]. Weight status by z-score and percentile was determined as per guidelines from the CDC using BMI for children age 2–20 years and Weight for Length (WFL) for those age <2 years [31,32]. Of the 2.008 evaluable children, 279 (14%) were obese and 117 (6%) 17 were underweight at diagnosis.

In Italy, 126 newly diagnosed pediatric cancer patients were included in the study of Triarico et al. [33]. For each patient, nutritional risk has been assessed with STRONGKids—a quick, reliable, and practical screening tool—to identify patients with risk of undernutrition [34,35]. Subsequently, anthropometric measurements—such as weight, height, BMI z-scores—were evaluated. The analysis showed a 100% rate of patients at risk of undernutrition at diagnosis. Respectively at diagnosis 90 patients (71.4%) presented a moderate risk of undernutrition (STRONGkids 2 or 3), whereas the other 36 (28.6%) were at high risk of undernutrition (STRONGkids 4 or 5). Sixteen patients (12.7%) presented mild undernutrition (BMI z-score from (−1)–(−1.9)), two patients (1.6%) presented moderate undernutrition (BMI z-score from (−2)–(−2.9)) and four patients (3.1%) showed severe undernutrition (BMI z-score ≤ −3).

In Poland, the authors of [36] studied the frequency of undernutrition and obesity at diagnosis. A study group of 734 patients with various diagnoses was enrolled. Patients were divided into groups depending on the type of neoplasms: ALL, acute non-lymphoblastic leukemia (ANLL), HL, non-Hodgkin lymphoma (NHL), neuroblastoma (NB), Wilms’ tumor, and mesenchymal malignant tumor (MMT). Body weight and height were measured, and BMI was calculated at the time of diagnosis. At cancer diagnosis moment, 21.5% (158) of patients were undernourished, 64.7% (475) weighed properly and 13.8% (101) were overweight. Height deficiency was observed in 8% (57) of the patients, of whom 10% (34) were boys and 9% (23) were girls. Both underweight and short stature were found in 2% (15) of the patients. Among diagnoses, there were no notable difference considering height deficiencies. Patients in the ALL group were overweight more often than the rest of the study group (Risk Ratio (RR) = 1.82, Confidence Interval (CI) 95% 1.26–2.63, *p* = 0.002)—18.6% of them were overweight. However, children with MMT were less susceptible to overweight than the rest of the patients (RR = 0.36, CI 95% 0.15–0.87, *p* = 0.021)—only 5.4% of them were overweight. Girls with ALL were undernourished more often than other patients (RR = 1.72, CI 95% 1.08–2.75, *p* = 0.03). There were no significant differences in the undernutrition/obesity frequency in other neoplasms groups.

In Australia, Small et al. [37] retrospectively reviewed the growth and NS—assessed by BMI—of children diagnosed with NB. One hundred fifty-four children were diagnosed with NB, while only 129 of them had length/height and weight measurements recorded at diagnosis. At that moment, almost a quarter—31 children—(24.0%) were classified as underweight indicating a high incidence of undernutrition, while the percentage of overweight patients was 11.6% (*n* = 15). There was no noteworthy difference in gender, age, or disease stage at diagnosis across children who were classified as underweight, normoweight or overweight.

It is therefore understood that weight and height are important measurements for assessing a child′s NS. According to Brinksma et al. [38], the evaluation of weight and height at the time of diagnosis in comparison with the measurements prior to diagnosis is particularly important in children who have recently been diagnosed with cancer. However, children who suffer from severe weight loss or lack of linear growth, but have what is considered appropriate weight and height parameters—between −2 and +2 standard deviation scores (SDS)—, may also be poorly nourished [38]. The authors studied a group of 95 patients, 45 (47%) of which were females. Children were diagnosed with hematological malignancies (57%), solid (26%) and brain (17%) tumors. At diagnosis, weight and height measurements were recorded and compared with the child’s own growth potential—authors used data collected in Primary Health Care Corporation (PHCC). Undernutrition was observed in 2% (2 out of 95), 4% (4 out of 95), and 7% (7 out of 95) of the children according to zWFA<-2 SDS, zHFA<-2 SDS, and z-scores for weight for height (WFH) <-2 SDS, respectively. However, when compared to their growth curves another 20–24% of children lost more than 0.5 SDS in WFA, HFA, and WFH z-score. In conclusion comparison of weight and height at diagnosis with data from growth curves indicated that—on average—children’s z-scores of weight and height at diagnosis were lower than predicted from their growth curves. Actually, more children were poorly nourished than weight and height at diagnosis indicated [38].

In the same country, Loeffen et al. [39] studied—amongst others—malnutrition at diagnosis within a heterogeneous childhood cancer population. The study sample consisted of 269 children with cancer, receiving treatment for various malignancies. BMI z-scores were used as indicator of NS. At the time of diagnosis, 14 children (5.2%) were classified as undernourished (BMI z-score < −2), 229 children (56.9%) were adequately nourished while 19 (7.1%) were over-nourished (BMI z-score > 2). Undernourished children showed poorer survival versus adequately nourished (hazard ratio (HR) = 3.63, 95% confidence interval (CI) = 1.52–8.70, *p* = 0.004).

According to published studies, the majority of data on NS of children with cancer at the time of diagnosis relates to undernutrition. Numerous studies include diverse measures and assessment methods, leading to a highly variable prevalence of undernutrition at diagnosis [1]. Table 1 summarizes the characteristics of eligible studies that assess nutritional status at the time of diagnosis.

The reported differences between studies are due to the fact that nutrition related problems—particularly the prevalence of undernutrition—depend on factors such as the timing of nutritional assessment. Nutritional assessment at diagnosis is often postponed in the context of many other procedures that may have a higher priority, some of which may even affect it [40]. In addition, there is no clinical “gold standard” to assess the NS [1]. The methods used make the criteria of malnutrition heterogeneous, as the process depends on the sensitivity and specificity of the parameters [8]. Furthermore, the time of cancer development is not the same for all diagnoses. If cancer develops more rapidly—e.g., hematological malignancies against solid tumors—the shortage of weight is lower, as there is not enough time to develop severe nutrition deficiencies [36]. Moreover, the reported differences as regards the prevalence of undernutrition are due to the composition of each study population regarding the types of malignancies. In the majority of studies, patients are categorized into hematologic, solid, and brain malignancies [3].

Nutritional assessment at the time of diagnosis, is probably the most appropriate time to prevent the deterioration of NS. Undernutrition worsens as the disease progresses, meaning that the longer the diagnosis is delayed, the higher the risk of undernutrition [41]. Therefore, the early diagnosis of undernutrition and early intervention should be a priority in all interdisciplinary oncological teams in an effort to solve at least part of the problem [24].

### 3.2. Nutritional Status during Treatment

NS at the time of diagnosis is an important factor which influences the response to the treatment, as well as the possibility of recovery [1]. However, malnutrition in pediatric patients with cancer is dynamic and development of impaired NS is commonly seen during subsequent treatment [42]. The adverse effects of nutritional problems during treatment, such as reduced tolerance of chemotherapy, alterations in drug metabolism, reduced immunity, increased risk of infection, and compromised QOL, have been established, however the quality of the evidence supporting each of these effects is variable [43].

Previous reports addressing the impact of weight on treatment-related toxicity (TRT) and event-free survival (EFS) in acute leukemia were limited by taking into account patient’s weight only at diagnosis [44,45,46]. As weight varies significantly during the treatment course of pediatric ALL [47], Orgel et al. [25] evaluated the effect of weight alterations on EFS and development of TRT, all along the treatment period in contrast to weight at diagnosis. A multitudinous group of children diagnosed with and treated for high-risk ALL was enrolled in the analysis. Orgel et al. [25] observed that only those children with constant underweight or obese status across intensive phases of treatment for high-risk ALL were at substantially higher risk for TRT occurrence, relapse, or death. Furthermore, for patients whose NS status-either obesity or underweight-was constant for > half of pre-maintenance therapy, the risk for future relapse or death was up to double compared with patients who remained normoweight during the treatment course. Contrarily, the risk of patients who began treatment obese or underweight and subsequently ended up normoweight/overweight, decreased to become comparable to being normoweight throughout. In addition, obese or underweight children were facing greater risk for specific toxicity profiles, an essential independent issue in efforts to decrease morbidity resulting from effective but toxic treatment protocols.

Paciarotti et al. [48] performed a prospective cohort study, aiming to determine both the prevalence of undernutrition and over-nutrition—overweight and obesity—and to detect critical changes in NS with reference to tumor type, treatments, and nutritional interventions. NS assessment combined several parameters—dietary intake, BMI centile, TSFT and MUAC—and was performed at diagnosis and at three months after treatment initiation. In terms of diagnosis, cohort was grouped in children with leukemia and in children with other types of cancer. Undernutrition prevalence—determined by BMI centiles—was highest among the “other cancers” group at diagnosis. The low BMI centiles were correlated with a higher prevalence rate of undernourished children in comparison to the anticipated undernutrition rate for the UK population [28]. On the other hand, the “leukemia” group, demonstrated excess BMI centiles at both time points and the prevalence of obesity was greater than the expected for the UK population. The BMI alterations, as time went on, followed the anthropometric variations. The “leukemia” patients had excess fat reserves during treatment course—measured by Upper Arm Fat Area (UAFA)—being 130% of standard at three-month time. The “other cancers” group had depleted fat stores, with UAFA values getting lower from 78% at diagnosis to 70% of standard at three months of treatment, suggesting a negative energy status existing prior to diagnosis. Consequently, both undernutrition and obesity are frequent disorders during the first phase of treatment for pediatric cancer with clear differences among cancer diagnoses.

There are several studies that present data on malnutrition in children with cancer, however little is known about the timing of under- and over-nutrition onset, as well as their respective causes. Brinksma et al. [49] intended to determine in which treatment phase NS deterioration occurred, and which factors lead to these alterations. A prospective cohort study of 133 newly diagnosed cancer patients with hematological malignancies, solid and brain tumors was performed. Anthropometric measurements and related date were recorded at admission and at three, six and 12 months after diagnosis. Despite initial weight loss at the beginning of treatment in patients with hematological and solid malignancies, BMI, and fat mass (FM) increased within three months by 0.13 SDS (*p* < 0.001) and 0.05 SDS (*p* = 0.021) respectively. Increase continued during the following months and resulted in a doubling of the number of over-nourished patients. Fat-free mass (FFM)—which was already low at diagnosis—remained low. During the entire study period about 17% of the patients were undernourished on the basis of low FFM. The most important changes took place within three months after diagnosis. Particularly WFA and BMI decreased at first, in patients with hematological malignancies and solid tumors, while tended to increase in patients with brain tumors. In a three–month period both WFA and BMI increased in all diagnoses, compared to the time of diagnosis. Furthermore, HFA decreased in all diagnostic groups, whereas MUAC increased. TSFT, % FM, and FM were higher, especially for patients with BT. FFM was constant and values were lower in patients with brain tumors in comparison to children with hematological malignancies and solid tumors. Furthermore, stagnation of growth in terms of height contributed to increase in BMI. Consequently, it is imperative for clinicians to comprehend that in order to prevent increase in BMI during treatment, weight should remain stable until growth in height continues.

Iniesta et al. [50] performed a prospective cohort study to examine the prevalence of malnutrition, NS alterations and factors contributing to nutritional disorders in Scottish pediatric cancer patients aged < 18 years. Clinical and nutritional data, as well as anthropometric measurements—MUAC and TSFT—were recorded at specific periods up until 36 months after diagnosis. The study population was grouped conforming to the wider definition of solid and brain tumors, hematologic malignancies, and other associated diagnoses. The prevalence of malnutrition—undernutrition, overweight and obesity—differed at various time points and among the anthropometric measurements. Overall, undernutrition was higher at diagnosis than at any other time whereas no patient was undernourished at the end, i.e., 30 and 36 months. In contrast, overnutrition increased over time. Particularly overweight was highest at 36 months and obesity was most prevalent at 30 months. As to diagnoses, patients with brain tumors and other associated diagnoses had the highest prevalence of overweight and obesity, even at the start of treatment. Contrarily, children diagnosed with solid tumors had the highest prevalence of undernutrition, followed by brain tumors and hematological malignancies during the first stages of treatment. In conclusion, the study highlights that children diagnosed with and treated for cancer are at high risk of undernutrition—notably during the first three months of treatment—and of over-nutrition at later stages. The most significant component contributing to undernutrition during the first three months of treatment was high treatment risk.

With regard to solid tumors, NB is one of the most common solid tumors in children [51]. Small et al. [37] wanted to examine retrospectively the BMI status of children treated for NB. One hundred and twenty-nine children diagnosed with NB were recruited in the study. Anthropometric measurements were collected at diagnosis as well as at various time points, up until five years after diagnosis. At diagnosis 24% of children were classified as undernourished and 11.6% were overweight. At six months after diagnosis, children in almost all disease stages showed a significant decrease in age and sex adjusted BMI. Subsequently, weight z-scores began to increase so that at 12 months’ time higher BMI z-scores were observed in children in all disease stages. Over the following four years, BMI z-scores either gradually changed or stabilized depending on the stage of the disease. Almost five years after diagnosis, the proportion of underweight children decreased to 8.7% while the proportion of children who were classified as overweight doubled to 28%. Even though low BMI values are common in children with NB—particularly at diagnosis and during treatment—the authors did not find any association between BMI and survival rates. Yet, the high proportion of overweight children at follow-up underscores the importance of nutritional interventions [37].

In India, Radhakrishnan et al. [26] conducted a study to look at the prevalence of malnutrition and to assess the impact of treatment on NS of pediatric cancer patients. A total of 295 pediatric patients were enrolled in the analysis. They were provided all meals and nutritional supplements by the hospital during their treatment duration. Data on WFA were available for 295 patients at diagnosis, 282 patients at midway through treatment, and 152 patients at the end of treatment. At diagnosis, undernutrition was seen in 44% patients, which increased to 46% midway during treatment, and decreased to 27% at the end of treatment (*p* = 0.0005). Even though undernutrition is a common problem in patients in resource-poor countries such as India, this study highlights that active nutritional intervention and education were able to significantly reduce the prevalence of undernutrition in patients at the end of treatment.

Nutritional support aims to reverse undernutrition seen at diagnosis, prevent undernutrition associated with treatment and promote weight and growth. There is no doubt that a poor nutritional state is a clear prognostic factor for treatment response and has an effect on the outcome of children with cancer [42].

### 3.3. Nutritional Status and Clinical Outcome

The presence of undernutrition correlates with a greater number of complications and relapses, as well as with decreased level of recovery [1,52]. Undernutrition can adversely affect the overall survival (OS), because it may reduce the tolerance to chemotherapy, increasing treatment-related morbidity (TRM) and decreasing EFS [21,39,53].

According to Barr et al. [43], two landmark retrospective studies were performed in the (CCG). Lange [54] conducted a study which included 768 children and adolescents with AML. Eighty-four patients (10.9%) of the study population underwent weight loss and 114 (14.8%) were overweight or obese, defined by BMI (≤10th percentile and ≥95th percentile respectively). Children with abnormal weight had remarkably worse survival than normoweight because of higher TRM rates.

Butturini et al. conducted the subsequent report, regarding >4000 children and adolescents with ALL. The CCG researchers focused on the effect of obesity, again as defined by BMI. The five-year EFS was poorer and the relapse rate higher in obese (*n* = 343,8%) compared to non-obese patients, but only in those aged 10 years and older. After these two landmark studies in pediatric leukemia, multiple analyses from international research centers have since described inconsistent associations between obesity and leukemia survival [25,45,55,56,57,58], raising uncertainty as to whether such a relation exists and, if so, to what extent.

Amankwah et al. [59] performed a meta-analysis which further complicated data interpretation through inclusion of a wide variety of leukemia types, therapeutic modalities, and differences in baseline survival rates between high- and low-income countries (LICs) [60,61]. The authors aimed to evaluate the association between BMI at diagnosis and pediatric acute leukemia mortality and relapse. An increased risk of mortality with a high BMI at diagnosis was observed (OS: HR = 1.30, 95% CI = 1.16–1.46 and EFS: HR = 1.46, 95% CI = 1.29–1.64). Sub-group analysis for ALL, the most prevalent form of pediatric acute leukemia, revealed a stronger association for both OS (HR = 2.25, 95% CI = 1.33–3.82, *p* = 0.002) and EFS (HR = 1.49, 95% CI = 1.30–1.71, *p* < 0.001). Overall a high BMI at diagnosis was associated with poor OS and EFS among pediatric acute leukemia patients [59].

In contrast to the previous review [59], Orgel et al. [62] included in their analysis a relatively uniform population from HICs in order to determine whether a higher BMI at diagnosis of pediatric ALL or AML is associated with worse EFS, OS, and cumulative incidence of relapse (CIR). As regards ALL, the authors observed poorer EFS in children with a higher BMI (RR = 1.35, 95% CI = 1.20–1.51) than in those with a lower BMI. A higher BMI was associated with significantly increased mortality (RR = 1.31, 95% CI = 1.09–1.58) and a statistically nonsignificant trend toward greater risk of relapse (RR = 1.17, 95% CI = 0.99–1.38) compared with a lower BMI. In AML, a higher BMI was significantly associated with poorer EFS and OS (RR = 1.36, 95% CI = 1.16–1.60 and RR = 1.56, 95% CI = 1.32–1.86, respectively) than was a lower BMI. However, other studies have reported different outcomes.

In the recent retrospective analysis, Saenz et al. [63] evaluated the association between overweight/obesity (BMI ≥ 85th percentile) at pediatric leukemia diagnosis and relapse or mortality. The study included 181 pediatric patients diagnosed with ALL, AML, and chronic myeloid leukemia (CML). The authors observed a statistically significant association between mortality and obesity status in unadjusted models that disappeared in both age- and sex-adjusted and multivariable-adjusted analysis. Analysis limited to ALL patients—the most common type of leukemia—showed no association between relapse or mortality and obesity status. As expected, analysis based on the small number of AML cases only, did not show any statistically significant association for relapse (HR = 3.93, 95% CI = 0.71–21.82, *p* = 0.12) or mortality (HR = 1.39, 95% CI = 0.31–6.27, *p* = 0.67) either. A meta–analysis combining these findings with those of previous studies was also performed. Concerning ALL overweight/obese patients, the meta-analysis revealed an increased mortality and relapse risk (HR = 1.79, 95% CI = 1.03–3.10) and (HR = 1.28, 95% CI = 1.04–1.57) respectively. Similarly, an association between obesity and increased risk of mortality was observed for AML patients (HR = 1.64, 95% CI = 1.32–2.04) [63].

Aldhafiri et al. [55] conducted a study in the UK, on a cohort of 1,033 patients. The authors found no evidence to support the association between overweight/obesity at diagnosis and childhood leukemia relapse [55]. These findings are consistent with previous studies as well. In the UK, Weir et al. [64] examined the effects of BMI at diagnosis on leukemia relapse in children (*n* = 1,025) and found no statistically significant association between obesity and relapse. Two more studies in the US [57,65] also failed to detect an association between obesity at diagnosis and risk of relapse in children with ALL. In Turkey, Karakurt et al. [66] did not observe any difference between mean BMI at diagnosis and relapsed or non-relapsed patients. The authors of [67] did not find any association between BMI at diagnosis and prognosis for children aged 2–9 years, but they observed a trend for improved outcome in overweight patients aged 10–17 years.

So far, data have been related to hematological malignancies, while studies evaluating the role of NS in pediatric solid tumors are lacking. Joffe et al. [68] conducted a study aiming to summarize data reporting on the association of NS and treatment-related outcomes—TRT, EFS, CIR and OS—in children and adolescents diagnosed with a solid tumor. Finally, 10 reports met the criteria and were included in the review. Up to 62% of patients were over- or undernourished at diagnosis [69]. Four out of 10 included studies identify abnormal BMI as a poor prognostic indicator in this group of patients [70,71,72,73]. Abnormal BMI was associated with worse OS in Ewing sarcoma (HR = 3.46, *p* = 0.022), osteosarcoma (HR = 1.6, *p* < 0.005), and there was a trend toward poorer OS in rhabdomyosarcoma (HR = 1.70, *p* = 0.0596). High BMI in osteosarcoma was associated with increased nephrotoxicity and postoperative complications. Regarding other included disease categories, NS was not a significant predictor of outcomes.

Iniesta et al. [74] aimed to evaluate the primary research on the prevalence of malnutrition in children with cancer and determine whether there are correlations between malnutrition and clinical outcomes. According to the authors [74], correlations between undernutrition and clinical outcomes remain unclear, with some researchers arguing that undernutrition is associated with worse outcomes [8,75,76,77] and others claiming that there are no such associations [64,78]. Undernutrition may be associated with higher mortality [8,77]. Yet, both studies referred to developing countries, so findings may have been affected by other factors regarding mortality. As regards undernutrition and relapse, two large studies [8,64] found no associations. Obesity in children with ALL was not associated with a decrease in EFS, when obese children were compared with normoweight children [65]. On the contrary, Butturini et al. [44] found that obesity at diagnosis independently predicted the likelihood of relapse in pre-teenagers and adolescents with ALL.

Moreover, the majority of research studies regarding the effect of malnutrition on infections and mortality have been conducted in homogenous populations including patients with one diagnosis. Loeffen et al. [39] investigated whether malnutrition is a prognostic factor for infection rates and survival, within a heterogeneous childhood cancer population. The authors of [39] showed the strong association between rapid weight reduction within the first three months of treatment and increased rate of Febrile Neutropenia (FN) episodes. A group 269 children diagnosed with and treated for cancer were enrolled in the analysis. During the first year after diagnosis, 332 admissions for FN were recorded. As regards the incidence of these episodes, there were no statistically remarkable difference between patients who were adequately nourished at diagnosis and patients who were under- or over-nourished. Nevertheless, BMI z-score decrease >1.0 (*n* = 13) and weight loss >5% of the initial body weight within the first three months after diagnosis, were strongly associated with FN episodes occurrence—(*p* = 0.010) and (*p* = 0.004) respectively. Regarding NS, survival was notably worse (*p* = 0.01) for undernourished children at diagnosis (*n* = 14) than for those who were adequately and over-nourished (*n* = 248).

Pribnow et al. [21] examined the correlations between NS and cancer type, TRM and EFS. A total of 282 patients diagnosed with Wilms tumor, ALL, AML, HL or BL were included in the study. Children diagnosed with Wilms tumor had the highest prevalence of undernutrition (85.7%), followed by children with BL (75%) and AML (74.3%). As regards TRM, 92.2% of patients experienced morbidity during the first three months of treatment whereas 84% of patients experienced severe morbidity. TRM in pediatric patients with cancer was associated with NS, as morbidity was greater in children with severe undernutrition than in those with adequate nutrition (*p* = 0.023). Another crucial finding in this study was the association between NS and severe infection, as infection is a major cause of mortality and was the second leading cause of death during the study period (22.9%). Particularly undernutrition was associated with severe infection (*p* = 0.033). In addition, undernourished patients had inferior median EFS (*p* = 0.049) and abandoned therapy more frequently (*p* = 0.015).

With regard to the increased risk of infections and their incidence, Triarico et al. [33] recently confirmed their association with nutrition related problems. In their retrospective study 126 newly diagnosed children 3–18 years old were included. Overall, 298 admissions for FN occurred during the first year after diagnosis. On average, children had two admissions for FN while 54 patients (42.9%) had ≥3 admissions for FN during the first year after diagnosis. A number of hospitalization for FN ≥3 was found in children moderately to severely undernourished at three and six months after diagnosis, in patients with weight loss ≥5% at three months, in the case of a weight loss ≥10% at six months and finally in patients with a BMI z-score decrease ≥1 at six months. Analyses of weight loss and BMI z-score decrease demonstrate that in a period of three and six months from diagnosis there was a three-fold increase of the rate of at least moderate undernutrition, from 4.7% to 14.3% and 13.5% respectively. Indeed, at three months 58 children (46%) underwent weight loss ≥5%. At six months, they were 63 (50%), while 28 of them (22.2%) had lost ≥10% of their body weight. Furthermore, weight loss ≥5% at three months and weight loss ≥10% at six months after diagnosis were remarkably associated with higher mortality. Mortality risk increased by 294% in patients who lost ≥5% of weight in a three-month period after diagnosis and by 110% in patients who reported weight loss ≥10% at six-month time.

Given the high prevalence of nutrition related problems during childhood cancer treatment and their impact on clinical outcomes, nutritional assessment should be mandatory from diagnosis and during treatment, in view of the possible manageable nature of this risk factor. Early adaptation of the NS screening for pediatric cancer patients could not only improve their survival, but also their QOL [33].

### 3.4. Nutritional Status and Health-Related Quality of Life

Malnutrition during treatment for childhood cancer has not only substantial clinical implications, but may also adversely affect a child’s QOL [79]. Until recently, QOL in children with cancer was unexplored [80]. In recent decades, cancer survival rates have increased thus emphasizing the importance of children’s personal needs. As a result, health-related quality of life (HRQOL) in childhood cancer patients has become a crucial issue in clinical practice [79]. HRQOL of children and adolescents is complex as children grow and develop. On the other hand, multimodal therapy that combines intensive chemotherapeutic protocols, surgery and radiotherapy induces many side effects which adversely affect children’s HRQOL [81].

Tsiros et al. [82] conducted a literature review on HRQOL in healthy, though obese children and adolescents. Findings support that being overweight/obese has an unfavorable impact on social and emotional functioning. In addition, other previous studies [83,84,85,86] have found that children diagnosed with and treated for malignancies had the lowest HRQOL, in comparison to healthy peers or children with other diseases. Broadly, it is considered that HRQOL in undernourished patients is lower when compared with adequately nourished patients [87] and that NS amelioration will lead to better HRQOL. However, the correlation between NS and HRQOL in childhood cancer patients had not been examined until 2015.

Brinksma et al. are the first to examine the association between NS and HRQOL across childhood cancer patients. Notably, they studied the association of undernutrition, over-nutrition, weight loss, weight gain with HRQOL in a heterogeneous group of children with cancer one year after diagnosis. In total, 104 patients (aged 2–18 years) diagnosed with hematological (43%), solid (33%), or brain malignancies (24%) participated in the study. Weight, height, and BMI were assessed and expressed as SDS. Furthermore, FFM and FM were calculated based on bioelectrical impedance analysis (BIA). The child- and parent-report versions of the PedsQL 4.0 Generic scale [86,88] and the PedsQL 3.0 Cancer Module [89] were used in order to measure generic and cancer-specific HRQOL. According to Brinksma et al. [79] nutrition related problems adversely affect HRQOL in childhood cancer patients. Undernourished as well as over-nourished children experienced poorer QOL than children who were well-nourished. Similarly, both noteworthy weight loss and weight gain led to worse HRQOL. Actually, impaired physical functioning prevailed in undernourished patients and in patients with weight loss. It is widely known that undernutrition and weight loss are associated with muscle mass deficit and muscle deficiency, resulting in fatigue [90]. Therefore, undernourished children did not have the vitality and muscle strength needed to involve themselves in physical activities. Furthermore, undernutrition adversely affected children’s social functioning. This finding can be justified by the pain, nausea, and tiredness these children encounter, which impair their ability to sufficiently participate in physical and social activities with their peers. As regards the psychosocial field, both over-nourished children and children with weight gain showed impaired functioning—when compared to adequately nourished patients—particularly in the emotional and cognitive sphere. They were more susceptible to feelings of fear, sorrow, and anger. Hence, they experienced more difficulties in interacting with others and they struggled to perform cognitive tasks—compared to adequately nourished patients with cancer.

These results have implications in clinical practice as they indicate the significance of adequate NS in children with cancer. Although the study could not demonstrate a causal relationship between nutrition related problems and HRQOL, undernourished and over-nourished patients experienced the lowest HRQOL across all cancer patients.

### 3.5. Nutritional Status during Survivorship

Advances in treatment have resulted in considerable improvements in survival rates of pediatric cancer. This success translates into a growing population of long-term survivors [91]. However, almost two-third of childhood cancer survivors (CCSs) will encounter at least one late effect while 40% of them are vulnerable to experience a disabling or potentially fatal condition even 30 years after diagnosis [92]. Even though late effects and chronic health conditions may be largely attributed to cancer and its treatment [93], the effects of NS that extend into survivorship put survivors at risk for numerous nutrition-related morbidities considering the pre-existing risk factors CCSs are facing [94]. There is a significant body of literature on NS among cancer survivors in childhood and adolescence after completion of treatment in HICs [43]. Special attention has been paid to ALL diagnosis, given its dominant prevalence in this age group worldwide [95] (Table 2).

Zhang et al. [96] performed a meta-analysis on the prevalence of obesity in pediatric ALL survivors in order to evaluate whether survivors are more likely to be obese than a reference population. Forty-seven studies met the inclusion criteria reporting on 9223 pediatric ALL survivors. Even though there was significant heterogeneity among studies, pediatric ALL survivors had considerably higher BMI than the reference population. There was a consistently high prevalence of overweight/obesity in both recent and long-term survivors regardless of patient- and treatment-related characteristics.

Although many studies have focused on obesity and the consequences of being overweight, it was unclear at which time period survivors experienced excessive weight gain. Zhang et al. [97] performed a retrospective cohort study of 83 children with ALL. BMI status was examined at various time points during and after treatment, as well as annually up to five years after treatment. The percentage of patients who were overweight or obese (BMI ≥ 85th percentile) doubled from 20% at diagnosis to about 40% after treatment completion [97]. Particularly, 26.7% of normoweight children became overweight/obese at the end of treatment and 36.1% were overweight/obese five years post-treatment. Among those who were overweight/obese at diagnosis, 81.3% and 66.7% remained overweight/obese at the end of treatment and five years post-treatment, respectively. The overall increase in BMI z-score from diagnosis to the end of treatment was associated with a more than threefold increased risk of being overweight/obese five years after treatment. The study reveals that patients with pediatric ALL were at risk of becoming overweight or obese early during treatment, while these changes in weight status remained throughout treatment and after treatment completion.

Zhang et al. [98] conducted a subsequent meta-analysis and come to similar conclusions. Findings demonstrated significant increase in mean BMI z-score and weight during treatment that persisted beyond treatment completion. Actually, unsound weight gain was prevalent in pediatric ALL patients regardless of receipt of cranial irradiation therapy (CRT), sex, and weight status [98].

Collins et al. [99] performed arm anthropometry to assess the NS in long-term survivors of ALL in childhood and adolescence, as BMI does not distinguish muscle from adipose tissue [106]. Seventy-five patients diagnosed with ALL at least before a decade, were enrolled in the study. According to BMI values only six survivors were undernourished and none of them severely. Twenty-five survivors were overweight/obese, while only six (8%) were actually obese. However, 15 (20%) survivors were obese—assessed by TSFT—and only 3% suffered from sarcopenia according to MUAC. As it results, malnutrition rates varied according to assessment methods performed.

Karlage et al. [100] reported similar inconsistencies regarding BMI and anthropometric measurements of body composition. Obesity rates varied between 40% determined by BMI, 62% by three site skinfolds and 85% by Dual-energy X-ray Absorptiometry (DXA). Even though skinfolds undervalued the percent body fat of CCSs when compared with DXA, they indicated higher sensitivity than BMI when used to assort survivors as obese or not obese. Particularly nearly 47% of males and 53% of females of the study population were misclassified as non-obese when assessed by BMI, which may result in CCSs not receiving appropriate nutritional support and guidance.

As regards to body composition, Marriott et al. [101] conducted a study focusing on skeletal muscle mass (SMM) alterations. The study included 75 long-term survivors—37 male and 38 female—diagnosed with ALL at least before a decade. Whole-body DXA scans were obtained, as well as measures of lean body mass (LBM), FM, and whole-body bone mineral content (BMC). According to fat mass index (FMI), the majority of females and two-thirds of males were overweight/obese—12% and 18% were obese respectively. On the basis of BMI, the percentages of overweight/obese became 35.3% for females and 31.3% for males—5.9% and 9.8% were obese respectively. The analysis of appendicular lean mass (ALM) showed that 50% of survivors ≤18 years old suffered from SMM deficit. Thirty-two survivors (43%) were identified with positive z-scores for FMI and negative scores for appendicular lean mass index (ALMI). Consequently, sarcopenic obesity prevails in long-term survivors of ALL, which puts them at risk of both excess body fat and insufficient SMM.

Apart from body composition alterations, changes in bone metabolism constitute extensive adverse late effects of cancer treatment. They represent a major cause of morbidity in the CCSs population through pain, fractures, decrease of BMD and chronic deterioration of bone function [107,108]. Molinari et al. [102] evaluated BMD and body composition in 101 patients treated for ALL, using bone densitometry and anthropometric data. As regards to NS, 22.8% of survivors were overweight and 15.8% were obese. As to body composition the LBM levels and BMC were higher in males, while FM levels and fat percentages were higher in females. The more time had passed from treatment completion until the time of the study, the higher the values of LBM, FM, percentage of fat and BMC. Among children and adolescents <20 years old (*n* = 79), three survivors (2.9%) had low BMD and 16 (15.8%) were classified as at risk for low BMD. Among survivors aged >20 years old, eight (7.9%) had osteopenia and none of them had osteoporosis. In comparison to the reference population most of the survivors had normal BMD values. However, the risk group—considered by the literature as with normal BMD—actually presented significantly lower bone mass values. Eventually, ALL survivors can regain lost bone mass during the post-treatment period. Yet, some of them will never achieve their higher BMD acquisition potential, presenting considerable bone deficit [109].

So far, most publications on the NS of CCSs focus on ALL. Wang et al. [103] systematically reviewed the prevalence of overweight and obesity of CCSs diagnosed with BT. As it emerges, evidence for weight gain and obesity among survivors of childhood brain tumors (SCBT) varies. Some studies report an increase [110,111], whereas others have shown no significant differences compared to healthy controls [112,113]. Among participants, survivors diagnosed with brain tumors other than craniopharyngioma and craniopharyngioma survivors were analyzed in separate groups in order not to overrate the prevalence of obesity in overall SCBT population, as patients diagnosed with craniopharyngioma are known to be at high risk of developing obesity [114,115]. According to BMI measures, overweight and obesity rates were similar for SCBT and general population—rate of combined overweight and obesity 42.6% and 40.4%, respectively. Yet, survivors had higher adiposity. As regards to craniopharyngioma, the participants had higher prevalence of obesity and combined overweight and obesity than SCBT and healthy controls. Specifically, overweight and obesity affected almost two-thirds of patients with craniopharyngioma.

Warner et al. [104] conducted a population-based study in order to evaluate the prevalence of undernutrition and overweight/obesity among 1060 adult CCSs of various diagnoses. The most prevalent diagnoses among female survivors were epithelial cancer (26.1%) and lymphoma (17.4%), while for males were lymphoma (23.8%) and central nervous system (CNS) tumors (16.3%). Considering all diagnoses, there were no differences between female or male survivors versus the age- and sex-matched comparison population, regarding the risk of being underweight or overweight/obese. However, according to BMI values 36% of females and 61% of male survivors were classified as overweight/obese. When further analyzed by cancer diagnosis, female epithelial survivors were less likely to be overweight or obese than the comparison population and only male CNS survivors had a slightly higher risk of being overweight or obese than the reference cohort.

Even though overweight, obesity, and MS have been broadly reported in the Western literature, data from developing countries are lacking. Prasad et al. [105] conducted a retrospective study and NS was assessed in a cohort of 648 Indian CCSs. At the time of the study 471 survivors were <18 years—child and adolescent survivors (CASs)—and 177 were 18 years or older. The prevalence of obesity, overweight, normal NS and undernutrition was 2.6%, 10.8%, 62.7%, and 28.8% for CASs while 0%, 8.5%, 62.7% and 28.8% for adult survivors, respectively. Regarding adult survivors, those >30 years old had higher prevalence of overweight compared to those <30 years old (22.2% vs. 6.9%, *p* = 0.004). None of them fit the strict criteria of MS, though 17 (9.6%) fit the lenient criteria which included overweight survivors as well as obese. As to CASs none participant fit the strict criteria for MS. However, 11 (2.4%) survivors had features of MS when the weight criteria were lenient. There was a higher prevalence of overweight/obesity between those diagnosed with ALL or BT (16.5% and 20.7%, respectively, *p* = 0.07) and the rest of survivors (13.6%). Overall the prevalence of obesity/overweight was lower in this cohort when compared to western literature. Yet, it is unclear whether these rates reflect the underlying undernutrition in developing countries such as India or the CCSs population of this study differ from their western counterparts. Despite the lower prevalence of overweight/obesity and MS in this cohort of survivors, CCSs remain at high risk for cardiometabolic complications.

Some of the risk factors for overweight, obesity and MS are modifiable and in the hands of CCSs themselves. There are guidelines for promoting health in cancer survivors, including encouraging healthy nutrition, physical exercise and avoiding high-risk behaviors [116,117]. Yet, dietary guidelines developed for cancer survivors—such as those developed by the American Cancer Society [118] and the World Cancer Research Fund/the American Institute for Cancer Research (WCRF/AICR) [119]—do not elaborate on CCSs. Furthermore, the long-term follow-up guidelines for CCS developed by the Children′s Oncology Group (COG) do not include cancer- and treatment-specific guidelines on nutrition [120].

Nevertheless, according to Brinkman et al. [121] healthy diet and physical exercise can moderate several late effects of cancer treatment, including obesity, hyperlipidemia, diabetes mellitus, cardiovascular disease, hypertension, and osteoporosis. Unfortunately, many CCSs do not meet the recommended dietary guidelines, with 54% of them exceeding their daily caloric requirements [122]. According to Zhang et al. [123], only 4%, 19%, 24%, and 29% of survivors follow the guidelines for vitamin D, sodium, calcium, and saturated fat intake, respectively. Nevertheless, nutritional intake in CCS has not been adequately studied [123]. Even though there are a few existing studies providing evidence that current dietary guidelines are not met [124,125,126,127], data are mainly derived from small groups of survivors or focus on specific cancer diagnoses—such as pediatric lymphoblastic leukemia. Zhang et al. [128] aimed to evaluate diet quality and dietary intake in a large cohort of 2570 adult CCSs. As regards diagnoses, leukemia was the most prevalent followed by lymphoma, embryonal tumor, sarcoma, and CNS tumor. The overall evidence emerges poor diet quality in CCSs, while older survivors had better diet quality than younger survivors did.

Unhealthy dietary behaviors have been associated with increased risk for health threatening conditions [94]. In addition, according to preliminary studies among CCSs, better quality diets may conduce to improved long-term health outcomes. There is developing evidence indicating that healthy dietary behaviors may reduce the risk of nutrition-related chronic conditions [94], thus it becomes essential to incorporate nutrition interventions and particularly dietary counseling into the clinical framework of survivorship care.

## 4. Discussion

Childhood cancer is an illness related to severe morbidity and mortality. The malignancy itself remains the main cause of death among childhood cancer patients [129,130]. Concurrently nutrition is a fundamental part of the pediatric cancer patients’ care. Adequate and appropriate nutrition is required to maintain optimal growth and development. Furthermore, adequate nutrition is likely to enhance survival outcome, reduce toxicity and improve QOL [53].

It has been widely recognized in literature that the NS of children diagnosed with and treated for cancer is likely to be affected at some point during the disease trajectory. Actually, for many childhood cancer patients, the early progression of the disease and the commencement of antineoplastic therapies can affect the NS, leading to malnutrition with many adverse consequences [8,74,131].

One of the most important findings of this review—that focuses on NS alterations that occur during the management of childhood cancer—is that the reported prevalence of malnutrition—undernutrition, overweight and obesity—varies between different types of cancer, different stages of the disease, type of treatment, as well as among studies, highlighting the complexity and diversity of this population. Children diagnosed with specific cancer types develop nutrition related problems more often than others. For instance, at diagnosis prevalence of undernutrition is higher in patients with solid tumors—especially Wilms tumor or neuroblastoma—and much lower in children with ALL and HL [4,11,14,21]. At the same time patients with brain tumors demonstrate high prevalence of overweight and obesity [50]. During treatment, children with solid tumors are more frequently nutritionally depleted, followed by brain tumors and hematological malignancies [49,74]. The detected differences in malnutrition among various diagnoses are inconsistent in LMICs, as delays in diagnosis and limited access to healthcare may lead to higher undernutrition rates, regardless of cancer type [21]. In addition, undernutrition is more often observed in high-risk diseases across all cancer types [21].

The majority of data that focus on NS of children with cancer at the time of diagnosis relates to undernutrition, the prevalence of which ranges from 10.8% [16] to 76% [23]. These reported differences between studies are due both to the stage of the disease at diagnosis and the parameters used to assess NS [8]. To date, there is a lack of consensus on the definition of malnutrition [1,28]. In addition, the criteria for NS assessment are heterogeneous [1,8,28]. Nutritional assessment is a process that depends on the sensitivity and specificity of the parameters performed [8]. Unfortunately, this procedure is often postponed in the context of many other procedures that may have a higher priority, some of which may even affect it [40]. Most studies refer to BMI as, it is widely used in clinical practice. Yet, it is not the most appropriate method for NS assessment because it does not measure body fat directly. Even though every method for clinical assessment of NS has restrictions, indicators such as MUAC, TSFT, and BIA provide more information regarding body composition changes that occur in paediatric cancer patients. Nonetheless, the diversity among different indicators of NS does not allow any straightforward comparisons among them. The prevalence of nutrition related problems depends not only on the methods and criteria used to assess the NS [8], the timing of the assessment [40] and the composition of the study population [3]—in terms of types of malignancies—but also on the socio-economic status. In general, undernutrition rates were much higher in LICs than in HICs [3,23,25].

Clearly, the NS at the time of diagnosis is an important prognostic factor that affects treatment response as well as the possibility of recovery [1]. However, nutrition related problems in pediatric cancer patients are dynamic and their development is usually observed during subsequent treatment.

Currently, the most relevant research is retrospective or cross-sectional. The few prospectively designed studies that have been published principally concern children diagnosed with hematological malignancies while most of them do not refer to the NS at all stages of treatment [74]. In addition, research has focused on the study of undernutrition during cancer treatment, while excessive energy intake or poor diet quality is being overlooked [74]. The adverse effects of undernutrition during treatment have been established [43]. Among others these include reduced tolerance to chemotherapy, changes in the metabolism of medicinal products, reduced immunity, increased risk of infections and degraded QOL [43]. However, the quality of data supporting each of these results varies.

Studies that investigate the association between NS at diagnosis and clinical outcome suggest that NS may affect cancer prognosis in children with cancer. Particularly, observed inferior survival was generally stable among studies [59]. The presence of undernutrition was associated with a large number of complications and relapses, as well as a reduced level of recovery [1,52,68]. On the other hand, excess weight gain and obesity negatively affected the response to treatment and led to reduced cure rates [1,4,52,53]. Yet, the correlations between nutrition related problems and clinical outcomes remain unclear, with some researchers claiming that they are associated to worse outcomes and others claiming that there are no such associations [53,74].

The NS status of children with cancer has not only significant clinical implications, but also can adversely affect the long–term development and health of survivors, including children’s QOL which as shown by the review remains underestimated [79]. There is a significant body of research on the NS of childhood and adolescent cancer survivors. Most studies focus on children with hematological malignancies—mainly in HICs—taking into account their predominant prevalence [43]. Furthermore, many reports confirm the impact of low or excessive body weight on survival, while collective evidence consistently shows poor diet quality in CCSs [128].

The lack of standard protocols and algorithms for assessment and treatment of nutritional problems, as well as limited in time dietary interventions are important factors that contribute to significant rates of malnutrition according to the literature. Meanwhile there is a lack of international specific dietary guidelines for children with cancer. Future scientific research should emphasize on proposing certain criteria that could assist the establishment of dietary instructions, such as cancer type, NS at diagnosis, treatment protocol, as well as children’s gender and age.

Nutritional assessment should be mandatory from diagnosis and during treatment, in view of the possible manageable nature of this risk factor [33]. Therefore, early assessment of NS and timely intervention should be a priority in all interdisciplinary oncology teams, in order to integrate nutritional counseling into the clinical framework of care in an effort to address at least part of the problem [24].

## 5. Conclusions

NS of pediatric cancer patients plays a crucial role during the disease trajectory. The malignancy itself and the progression of the disease cause NS alterations, leading to malnutrition. In addition, the commencement of antineoplastic therapies affects energy balance with many negative consequences.

Malnutrition—undernutrition, overweight, and obesity—is linked to adverse outcomes from diagnosis to long-term survivorship. NS at the time of diagnosis is an important prognostic factor that affects treatment response and the possibility of recovery. The impact of impaired NS on clinical outcome and cancer prognosis is related to treatment intolerance due to nutrient deficiency and immune incompetence. Increased risk of infection and alterations in drug metabolism lead to delays and treatment cessation that result in higher relapse rates and lower survival rates. In addition, undernutrition during treatment correlates to a greater number of complications, increasing TRM and decreasing EFS. Yet, correlations between NS alterations and clinical outcomes remain unclear. Nutrition related problems can also adversely affect the long-term health of survivors, including children’s HRQOL. The effects of NS that extend into survivorship put survivors at risk for numerous nutrition-related morbidities.

Given the high prevalence of malnutrition during childhood cancer and as NS represents a modifiable risk factor, nutritional assessment should be mandatory from diagnosis, during treatment and subsequently. There are several methods for the clinical assessment of NS and each one of them has limitations and constraints. Among those performed in clinical practice MUAC, TSFT and BIA provide more information concerning body composition changes than BMI does. Nonetheless, the diversity among different indicators of NS prevents us from extracting safe results regarding the most suitable one. Ideally, the most appropriate indicator is the one that would not allow a malnourished child to remain underdiagnosed.

As regards pediatric oncology, advances in treatment and follow-up care are significant. However, there is still a lack of international specific dietary guidelines for children with cancer. Hopefully, future scientific research should emphasize establishing cancer- and treatment-specific guidelines for nutrition. Early monitoring and adaptation of pediatric cancer patients’ NS as well as timely nutritional intervention could improve their treatment response, their clinical outcome, their survival, but also their QOL.

## Figures and Tables

**Table 1:** Characteristics of listed studies that assess NS at diagnosis.

Author, Year (Location)	Study Design	Patients (*N*)	Diagnosis	Assessment Method	Nutritional Related Problems
Maia Lemos et al., 2016 [16] (Brazil)	Cross-sectional observational study	1154	Various diagnoses	BMI ^1^, TSFT ^2^, MUAC ^3^, AMC ^4^	Undernutrition
Villanueva et al., 2019 [19] (Guatemala)	Retrospective cohort study	1060	Hematological Malignancies Solid tumors Brain tumors	TSFT, MUAC, ALB ^5^ levels	Undernutrition
Yoruk et al., 2018 [20] (Istanbul)	Prospective observational cohort study	74	Hematological malignancies Solid tumors	BMI, TSFT, MUAC, WFA ^6^, HFA ^7^	Malnutrition
Pribnow et al., 2017 [21] (Nicaragua)	Retrospective study	282	ALL ^8^, AML ^9^, HL ^10^, BL ^11^, Wilms tumors	BMI, TSFT, MUAC, ALB levels	Undernutrition
Peccatori et al., 2018 [22] (Nicaragua)	Intervention study	104	Hematological malignancies Solid tumors	TSFT, MUAC, Height, Weight	Undernutrition
Shah et al., 2014 [23] (India)	Retrospective observational study	1693	Various diagnoses	BMI, TSFT, MUAC, ALB levels	Malnutrition
Radhakrishnan et al., 2015 [26] (India)	Retrospective study	295	Various diagnoses	WFA	Malnutrition
Orgel et al., 2014 [25] (USA)	Retrospective cohort study	2008	High-risk ALL	BMI	Malnutrition
Triarico et al., 2019 [33] (Italy)	Retrospective study	126	Hematological malignancies Solid tumors CNS ^12^ tumors,	STRONG_Kids_ score	Undernutrition
Połubok et al., 2017 [36] (Poland)	Retrospective cohort study	734	ALL, ANLL ^13^, HL, NHL ^14^, NB ^15^, MMT ^16^, Wilms tumors	BMI	Malnutrition
Small et al., 2015 [37] (Australia)	Retrospective review	154	NB	Height, Weight, BMI	Malnutrition
Brinksma et al., 2015 [38] (The Netherlands)	Retrospective study	95	Hematological Malignancies Solid tumors Brain tumors	Height, Weight	Growth alterations
Loeffen et al., 2015 [39] (The Netherlands)	Retrospective study	269	Hematological Malignancies Solid tumors Brain tumors	ΒΜΙ	Malnutrition

^1^ Body Mass Index (BMI), ^2^ Triceps Skinfold Thickness (TSFT), ^3^ Mid–Upper Arm Circumference (MUAC), ^4^ Arm Muscle Circumference (AMC), ^5^ Serum albumin (ALB), ^6^ Weight for Age (WFA), ^7^ Height for Age (HFA), ^8^ Acute Lymphoblastic Leukemia (ALL), ^9^ Acute Myeloid Leukemia (AML), ^10^ Hodgkin Lymphoma (HL), ^11^ Burkitt Lymphoma (BL), ^12^ Central Nervous System (CNS), ^13^ Acute Non–Lymphoblastic Leukemia (ANLL), ^14^ Non–Hodgkin Lymphoma (NHL), ^15^ Neuroblastoma (NB), ^16^ Mesenchymal Malignant Tumor (MMT).

**Table 2:** Characteristics of listed studies that assess NS in CCSs.

Author, Year (Location)	Study Design	Survivors, N/(Control)	Diagnosis	Assessment Criteria	Nutritional Related Problems
Zhang et al., 2014 [96] (USA)	Systematic Review and Meta-analysis	9223	ALL	BMI	Overweight/obesity
Zhang et al., 2014 [97] (USA)	Retrospective cohort study	83	ALL	BMI	Overweight/obesity
Zhang et al., 2015 [98] (USA)	Systematic Review and Meta-analysis	1791	ALL	BMI	Overweight/obesity
Collins et al., 2017 [99] (Canada)	Cross-sectional cohort study	75	ALL	BMI Arm anthropometry	Malnutrition
Karlage et al., 2015 [100] (USA)	Longitudinal cohort study	1361	Various diagnoses	BMI DXA ^1^ Anthropometry	Overweight/obesity
Marriott et al., 2018 [101] (Canada)	Cross-sectional study	75	ALL	LBM ^2^ FM ^3^ Whole-body BMC ^4^	Overweight/obesity Sarcopenic obesity
Molinari et al., 2017 [102] (Brazil)	Cross-sectional study	101	ALL	Bone densitometry Anthropometry	Overweight/obesity BMD ^5^
Wang et al., 2018 [103] (Canada)	Systematic Review and Meta-analysis	2032	BT ^6^	BMI FM	Overweight/obesity
Warner et al., 2014 [104] (USA)	Population-based study	1060/(5410)	Various diagnoses	BMI	Malnutrition
Prasad et al., 2015 [105] (India)	Retrospective cohort study	648	Various diagnoses	BMI	Malnutrition MS ^7^

^1^ Dual-energy X-ray Absorptiometry (DXA), ^2^ Lean Body Mass (LBM), ^3^ Fat Mass, ^4^ Bone Mineral Content (BMC), ^5^ Bone Mineral Density (BMD), ^6^ Brain Tumors, ^7^ Metabolic Syndrome (MS).
